# An unusual case of the syndrome of cervical rib with subclavian artery thrombosis and cerebellar and cerebral infarctions

**DOI:** 10.1186/1471-2377-12-48

**Published:** 2012-06-28

**Authors:** Mirza Jusufovic, Else Charlotte Sandset, Trine Haug Popperud, Steinar Solberg, Geir Ringstad, Emilia Kerty

**Affiliations:** 1 Department of Neurology, Oslo University Hospital, Oslo, Norway; 2 Department of Cardiothoracic and Vascular Surgery, Oslo University Hospital, Oslo, Norway; 3 Department of Radiology and Nuclear Medicine, Oslo University Hospital, Oslo, Norway; 4 Institute of Clinical Medicine, University of Oslo, Oslo, Norway

**Keywords:** Subclavian artery/pathology, Cervical Rib syndrome/complications, Arterial thoracic outlet syndrome, Stroke/complications

## Abstract

**Background:**

Cerebellar and cerebral infarctions caused by the syndrome of cervical rib with thrombosis of subclavian artery are very unusual.

**Case presentation:**

We report the case of a 49-year-old male patient with a right cervical rib compression leading to subclavian arterial thrombosis and both cerebellar and cerebral infarctions secondary to retrograde thromboembolisation. Follow-up imaging revealed partial resolution of the thrombosis after combined anti-coagulant and anti-platelet therapy. The cervical rib and first costa were surgically removed to prevent additional events.

**Conclusion:**

Cervical rib vascular compression should be promptly diagnosed and treated in order to avoid further complications, including cerebrovascular ischemic events.

## Background

The presence of a cervical rib was already mentioned in the works of Galen in the second century A.D, and in 16th century by Vesalius [1]. Arteriothromboembolic events due to cervical rib compression of subclavian artery are rare. The commonest source of cerebral thromboembolism is artery-to-artery embolism from heart or proximal circulation.

Symonds reported in 1927 two cases with an initial history of symptoms of right upper extremity claudication due to a right cervical rib, followed later by acute left hemiplegia [2]. He concluded that pressure of cervical rib upon the right subclavian artery led to subclavian thrombosis with right upper extremity embolisation and finally to embolic occlusion into the right middle cerebral artery [2].

In more recent literature, the term thoracic outlet syndrome (TOS) was introduced by Peet in 1956 to put together upper limb symptoms arising from neurovascular compression in the interscalene triangle [3]. TOS is classified into three categories according to neurovascular anatomy: arterial (subclavian artery, <1%), venous (subclavian vein, 4%–6%), and neurogenic (brachial plexus, 94%–97%).

Cerebral infarction or TIA caused by cervical rib-associated arterial compression is unusual and very few cases have been described [2,4-10]. In a study of 30 surgically decompressed instances of vascular TOS in 25 patients, only one patient suffered a transient ischemic attack (TIA) [11]. Among these, 22 cases had subclavian artery involvement. Cervical ribs (73%) were found to be the commonest predisposing factor for subclavian artery thrombotic disease in this study [11]. One of the most important arterial pathologic changes caused by cervical rib in arterial TOS are partial or complete subclavian artery thrombosis or aneurysm [12].

We present the history of a 49-year-old male with a complete subclavian arterial thrombosis and both cerebellar and cerebral infarctions secondary to a right cervical rib and retrograde thromboembolisation.

## Case presentation

A 49-year-old man was referred to us with a 3-day history of headache, pain and fluctuating weakness in the right arm, and a one-day history of acute dizziness, unsteadiness, and transient loss of vision in the right hemifield. He had hypertension, dyslipidemia, and 27-pack-year history of smoking. Six months earlier he was admitted to a local hospital due to pain and weakness when elevating his right arm. MR angiography showed a right brachial artery occlusion. Conservative treatment with aspirin 75 mg was initiated and the arm symptoms diminished.

Upon arrival at our hospital, the patient stated that the pain in the right arm was exacerbated by elevation, such as sleeping with his arms overhead. There was no history of neck trauma. His additional medications included irbesartan in combination with hydrochlorothiazide 300/25 mg and simvastatin 20 mg.

Blood pressure was not measurable and there was no detectable pulse in the right upper limb. On the left side, the blood pressure was 147/83 mmHg with a regular radial pulse of 72 beats per minute. Apart from left-sided hyperreflexia without Babinski sign, general medical and neurological examinations were unremarkable. Biochemical and hematological blood samples were normal.

**Figure 1:**
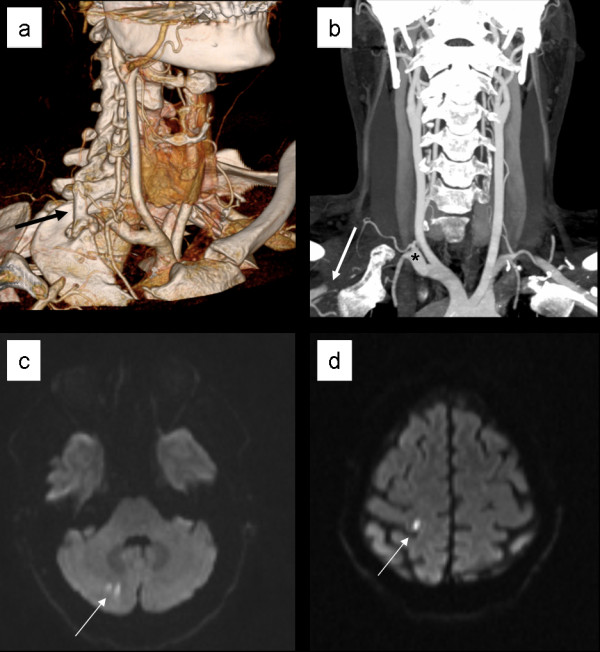
**Cervical rib (A) causing subclavian arterial thrombosis (A, B) and cerebellar (C) and cerebral infarctions (D).** Computed tomography angiography with surface rendering demonstrates a cervical rib (**A** black arrow) causing subclavian arterial thrombosis on the right side. The right clavicle has been subtracted from the image. Reformation with maximum intensity projection of the same volume. The right subclavian artery (**B** asterisk) is occluded proximal to the thoracic outlet and refilled with contrast by collateral vessels before entering the axilla (white arrow). Diffusion imaging (B1000) reveals punctuate foci of acute infarctions in the right cerebellum in **C** and posterior right frontal lobe in **D** (white arrows).

CT angiography showed a right-sided subclavian thrombus extending retrograde into the right carotid artery bifurcation and into the proximal segment of the vertebral artery, consistent with artery thrombosis (Figure 1A, B). The aortic arch was without signs of dissection or atherosclerosis. Brain MRI showed acute/subacute infarctions within the right cerebellum and in the superior parietofrontal lobe (Figure 1C, D), suggestive of emboli originating from the occluded subclavian artery. Imaging also showed bilateral cervical ribs (Figure 1A) with the right longer than the left, leading to reduced space between the cervical rib and anterior scalene muscle and compression of the right subclavian artery. Other possible explanations for cerebral thromboembolism were excluded by normal thrombophilia screening and transoesophageal echocardiography. Carotid Doppler examination showed that clots in the carotid were from propagating subclavian thrombus and were not anchored by plaque. Vessel wall inflammation and malignancy-predisposing thrombosis were excluded by 18 F-fluorodeoxyglucose positron emission tomography/computed tomography (PET/CT).

Based on the suspicion of cerebral thromboembolism originating from the occluded subclavian artery, low molecular weight heparin was initiated at 10000 units twice a day, followed by warfarin (target International Normalized Ratio 2,0-3,0).

CT angiography performed twice over a period of 3 months following the initiation of anti-coagulation showed partial recanalisation in all affected arteries. Thrombus extension retrograde into the right carotid artery bifurcation was not present at the second CT angiography prior to surgery. Four months after the cerebral thromboembolism the patient underwent resection of the right cervical rib and first costa through a transaxillary approach. The cervical rib was attached to the upper surface of the first costa by a pseudoarticulation. Both the surgery and postoperative period were uneventful, and the combined anti-coagulant and anti-platelet therapies were continued postoperatively.

## Conclusions

Our patient presented for six months with intermittent right upper extremity claudication due to pressure of cervical rib upon the right subclavian artery leading to thrombosis before cerebral thromboembolism. He was admitted to a local hospital six months before the recent cerebral infarction when thrombosis of the brachial artery was recognized. This is unusual in 49-year-old man. With a brachial artery thrombus, one should look proximally at the subclavian artery and regard the brachial artery thrombus as probably an embolus from the subclavian. At that time (six months ago) a cervical rib and subclavian artery occlusion should have been looked for and eventually repaired.

The first recognition that the syndrome of cervical rib with subclavian artery thrombosis could be complicated by combination of right upper extremity and cerebral thromboembolism was made by Gould in 1884 [9] and again in 1887 [8]. In Gould’s case, a 19-year-old male was described with “sickening pain” in his right hand [9], and an acute left arm paresis occurring three years later [8]. The pathogenesis was assumed as a progressive obliteration of the brachial artery, although “there was noticed a great prominence of the right subclavian artery above the clavicle” and “a bony mass springing from each side of the lower two cervical vertebrae, larger on the right than the left side” [8,9]. X-ray documentation of this compressive phenomenon was reported by Hoobler in 1942, who demonstrated bilateral cervical ribs in a patient with subclavian arterial thrombosis and cerebrovascular accident [10].

More recently, in a systematic review, Yamaguchi et al. reported ten patients with cerebral embolism from subclavian artery thrombotic pathology caused by a cervical rib or the first rib anomaly [13]. In a study of 120 young stroke patients, retrograde embolism due to a right cervical rib-associated arterial lesion was the reported cause in only one patient [14]. Retrograde propagation of subclavian thrombus can also involve the innominate artery [6].

Cervical rib represents an anomalous development of a rib, usually from the seventh cervical vertebrae [15], and may be associated with spinal anomalies elsewhere. The embryologic formation of cervical ribs is attributed to a conflict between forming ribs and plexuses [16]. In the present case, the most likely predisposing factor of subclavian thrombosis was the presence of this anomaly.

It has been reported that a cervical rib is present in less than 1% of the population [17] . They are asymptomatic in 90% of the cases [11] and are found twice as frequently in females than in males (68% versus 32%, respectively) [18]. Durham et al. found that 16 (73%) of 22 patients with subclavian artery compression had cervical ribs, and in five (31%) of these, bilateral cervical ribs were present [19]. In addition, soft tissue anomalies, such as scar tissue after neck-shoulder trauma and clavicle trauma, may also predispose an individual to subclavian artery compression [11]. Cervical ribs are recognised as complete or incomplete types [1]. Only complete cervical ribs have been reported to produce vascular symptoms [17,20].

Pain in the arm and hand is the prevailing symptom in subclavian artery compression, which typically presents in young, otherwise healthy patients with vigorous shoulder activity. The syndrome of occlusion is more common in athletes and baseball pitchers, golfers, and cricket bowlers in whom arm motions encourage contact with the artery and rib [6,21].

Common findings on physical examination consist of a pulseless, pale and cold distal upper limb. In order not to delay the diagnosis and correct treatment of arterial compression, the clinician must differentiate such arm ischemia from Raynaud syndrome, vasospastic disorders, distal small-artery obstructive disease, or proximally large-artery occlusions which may result in this symptom complex.

The lumen of the subclavian artery becomes constricted owing to the compression of the base of interscalene triangle in which the subclavian artery pass [17,22]. Chronic arterial spasm induced by the pressure of the cervical rib may increase flow velocity and shear stress at the affected arterial wall. These mechanical factors may damage the intima and trigger clotting, followed by thrombus formation, which in case of retrograde flow may break off to lodge in the cerebral region. Further arterial compression with increase in symptoms can occur during rotation and elevation of the arm [6,21,23].

In the present case, ischemia in both vertebrobasilar and carotid distribution were consequences of a thromboembolic migration from a bidirectional subclavian thrombus. In most reported cases of this unusual syndrome, the thrombosis occurred in the right subclavian artery and cerebral infarctions were caused by propagation of a thrombus through the carotid artery. Predilection of thromboembolism in the right cerebral and not cerebellar hemisphere is explained by the fact that only on that side the common carotid arises with the subclavian from the innominate [10]. This route of embolisation is also explained due to the larger caliber of and less vascular resistance in the common carotid than vertebral artery [13]. However, cerebellar infarction has been reported to occur if there is an extension of subclavian thrombus into the vertebral artery [24].

The syndrome of cervical rib with subclavian artery thrombosis is difficult-to-treat disorder. Decompression and vascular procedure is indicated when there is a failure to improve on conservative therapy [22] or where disability is manifested [17]. In our case, removal of the right cervical rib was deferred in order to prevent collapse of the subclavian thrombus and additional embolisation to the brain. The patient did not have any vascular sequelae and was initially treated with combined anti-coagulant and anti-platelet therapy and followed at regular intervals. He underwent resection of the right cervical rib and first costa through a transaxillary approach four months after the cerebral thromboembolism. CT angiography showed partial recanalisation in all affected arteries prior to rib resection, and it was therefore not considered necessary with reconstructive vascular procedure.

In conclusion, cerebellar and cerebral infarctions due to the syndrome of cervical rib with subclavian artery thrombosis are unusual, however, when present, the consequences for the brain tissue involved are serious. Surgical removal, instead of conservative treatment, should be considered in a patient with subclavian artery compression due to a cervical rib to prevent additional embolic events.

## Consent

Written informed consent was obtained from the patient for publication of this case report.

## Competing interests

The authors have nothing to disclose.

## Authors’ contributions

All authors contributed equally to this work. All authors read and approved the final manuscript.

## Pre-publication history

The pre-publication history for this paper can be accessed here:

http://www.biomedcentral.com/1471-2377/12/48/prepub
